# The effect of reading engagement on scientific literacy – an analysis based on the XGBoost method

**DOI:** 10.3389/fpsyg.2024.1329724

**Published:** 2024-02-14

**Authors:** Canxi Cao, Tongxin Zhang, Tao Xin

**Affiliations:** Collaborative Innovation Center of Assessment for Basic Education Quality, Beijing Normal University, Beijing, China

**Keywords:** scientific literacy, reading engagement, XGBoost, SHAP, PISA2018

## Abstract

Scientific literacy is a key factor of personal competitiveness, and reading is the most common activity in daily learning life, and playing the influence of reading on individuals day by day is the most convenient way to improve the level of scientific literacy of all people. Reading engagement is one of the important student characteristics related to reading literacy, which is highly malleable and is jointly reflected by behavioral, cognitive, and affective engagement, and it is of theoretical and practical significance to explore the relationship between reading engagement and scientific literacy using reading engagement as an entry point. In this study, we used PISA2018 data from China to explore the relationship between reading engagement and scientific literacy with a sample of 15-year-old students in mainland China. 36 variables related to reading engagement and background variables (gender, grade, and socioeconomic and cultural status of the family) were selected from the questionnaire as the independent variables, and the score of the Scientific Literacy Assessment (SLA) was taken as the outcome variable, and supervised machine learning method, the XGBoost algorithm, to construct the model. The dataset is randomly divided into training set and test set to optimize the model, which can verify that the obtained model has good fitting degree and generalization ability. Meanwhile, global and local personalized interpretation is done by introducing the SHAP value, a cutting-edge machine model interpretation method. It is found that among the three major components of reading engagement, cognitive engagement is the more influential factor, and students with high reading cognitive engagement level are more likely to get high scores in scientific literacy assessment, which is relatively dominant in the model of this study. On the other hand, this study verifies the feasibility of the current popular machine learning model, i.e., XGBoost, in a large-scale international education assessment program, with a better model adaptability and conditions for global and local interpretation.

## 1 Introduction

Scientific Literacy (SL) refers to the scientific nature of various forms of literacy in science, English, and technology and is relatively broad in scope (Roberts, 2013). Its development is essential not only for young people wishing to pursue a career in the sciences (e.g., physics, astronomy, etc.), but also for a citizen wishing to have a good life (Trefil and Hazen, 2007), and individuals need to possess a certain level of scientific literacy to be able to participate fully in society as a member of it (Cromley, 2009). In terms of assessment, in recent years, the assessment of core literacy, including scientific literacy, has formed a mature system, such as the Program for International Student Assessment (PISA), Trends in International Mathematics and Science Study (TIMSS), the International Mathematics and Science Study (IMSS), the International Mathematics and Science Study (IMSS), and the International Mathematics and Science Study (IMSS). These assessment programs are very comprehensive in their collection of educational information, which not only serves as a value guide, but also provides educational researchers with new perspectives on the educational process. In science literacy research, researchers can use the assessment programs to explore how individual student characteristics, teacher instruction, school management, and other factors affect student performance.

Existing research data suggest that there is a positive correlation between Reading Literacy (RL) scores and science literacy scores at all three levels: individual, school, and national (Cromley, 2009; Caponera et al., 2016). For the creation of this relationship between the two, researchers have pointed out that reading as an essential competency is present throughout learning activities. In the information age, which emphasizes the need for independent learning and lifelong learning for all-round development, the scientific information and channels for learning science that individuals can access on a daily basis are richer and more diversified than in the past, but they are also mixed with a large amount of irrelevant information. One of the concerns of educational researchers is how to search, screen, locate and acquire effective information in the sea of information, and then correctly interpret the information to form personal opinions and construct a cognitive system. In the process of acquiring, screening, and internalizing information, individuals can develop scientific literacy, but the whole process cannot be separated from the support of reading literacy, and with the increase of individual developmental needs, there are higher requirements for reading literacy. PIRLS points out that the fourth grade, that is, around the age of 9, is a key transition period for students’ reading development, and that students “learn to read” before fourth grade, and after fourth grade learn by reading (Tong et al., 2014). In short, reading is a powerful channel for promoting students’ ability to construct conceptual understanding, support inquiry, and develop scientific habits of mind (Wellington and Osborne, 2001; Yore and Treagust, 2006).

Whether it is reading literacy or scientific literacy, when viewed from the perspective of educational practice, they are more often presented as a kind of outcome or educational output. When thinking about how reading literacy acts on scientific literacy, it is not possible to put the laws of education on the ground of educational practice if we simply stay in the relationship between the two, and we need to take a step back and start from the examination of the factors related to reading literacy, such as basic reading skills, good reading attitudes, effective reading strategies, and the importance of reading literacy. We need to take a step back and look at the factors related to reading literacy, such as basic reading skills, good reading attitudes, effective reading strategies and critical thinking. Reading Engagement, which refers to reading activities in which individuals exhibit positive behaviors (e.g., actively seeking opportunities to read) and purposeful cognitive processes (e.g., the use of cognitive strategies), as well as emotionally profound experiences (e.g., obtaining pleasurable feelings) (Guthrie and Klauda, 2014), is intertwined with these three different factors (In the PISA framework, OECD identified reading engagement as the student characteristic most associated with reading literacy performance) (Kirsch et al., 2002), a concept that is rich in meaning and involves multiple aspects of knowledge and emotion, making it the best entry point for examining the relationship between reading literacy and scientific literacy. By analyzing the student characteristics of reading literacy, it is found that good reading habits and the flexible use of reading strategies have a profound impact on the acquisition of knowledge and skills in various areas, and a high level of reading commitment is conducive to the development of students in other areas (Britt et al., 2014).

Research on the impact of reading on scientific literacy is still mainly focused on scientific reading activities and key elements related to reading ability, such as reading interest and reading strategies. It is certain that students’ scientific literacy is closely related to scientific reading activities, and that effective scientific reading activities help students accumulate scientific knowledge and thus improve their scientific literacy (Fang and Wei, 2010). Effective science reading activities not only require the quantity of reading, but also the key elements of reading literacy such as reading interest and cognitive strategies, which are positively correlated with students’ scientific literacy to different degrees (O’Reilly and McNamara, 2007; Ozuru et al., 2009). Under the field of educational psychology, there are strong associations between reading-related concepts related to reading content and behavioral, cognitive, and affective attitudes during the reading process. Taking reading strategies as an example, students who mastered good strategies tended to excel in terms of reading duration and reading variety, and vice versa. Existing research on the relationship between reading and scientific literacy focuses on a few elements or starts directly from the concept of reading ability, which often tends to simplify the complex network of relationships and even leads to some one-sided interpretations, and it is necessary to adopt more comprehensive concepts and examine the impact of reading on the field of science from a more holistic perspective.

Reading engagement covers behavioral, cognitive and emotional factors, covering individual cognitive and non-cognitive processes, which is a relatively comprehensive and integrated concept, and reading engagement has a significant impact on the reading literacy of the youth group in the past research has been sufficient theoretical arguments and empirical evidence, through the analysis of international assessment data such as PISA and the use of self-developed scales for the investigation found that students with high reading engagement levels are able to perform better in the science field. Students with a high level of reading engagement are better able to use strategies, mobilize executive functions and thus gain a deep understanding of the text (Guthrie and Wigfield, 2000; Wigfield et al., 2008; Beer, 2010; OECD, 2010). In instructional experiments for college students, it has been demonstrated that interventions and instruction on reading engagement have some feasibility in improving students’ reading literacy, and is an indicator that can change in a short period of time (McNamara, 2017). Most of the existing research on the impact of reading engagement is still focused on the field of reading, which can be usefully explored by migrating the research on reading engagement to other subject areas.

In terms of data analysis methods, previous studies are still dominated by the establishment of traditional classical mathematical and statistical models. For large-scale international assessments such as PISA, which cover a wide range of variables, there are three levels of data from students, teachers, and schools in terms of subjects, and there are cognitive (e.g., beliefs, attitudes, etc.) and non-cognitive factors (e.g., ICT resources, socio-economic conditions, etc.) in terms of nature, and the complexity of the variable situation puts a high demand on the data analyzing tools; and the algorithms of regression modeling adopted by most of the researches or the algorithms based on the variance–covariance matrix-related algorithms (e.g., structural equation modeling, etc.), these algorithms based on statistical inference share some common problems, such as the need for restrictive *a priori* assumptions, the limited expressiveness of the model in the presence of a large number of variables, and the limited presentation of nonlinear relationships (Witten and Frank, 2002; Martínez Abad and Chaparro Caso López, 2017).

In recent years, the use of data mining machine learning techniques has yielded a number of satisfactory results in both sociology and educational assessment, such as the use of the GBRT family of algorithms to explore the key influences on loneliness among older adults, and the use of decision trees to explore factors affecting school effectiveness (Aksu and Güzeller, 2016; Gabriel et al., 2018; Martínez-Abad et al., 2020). In this way, the use of machine learning techniques for the analysis of educational assessment data is a good choice, with certain advantages in large-scale data analysis, not only to discover the more hidden valuable information carried by the data itself, but also because the main techniques of data mining, such as clustering, association rules or decision trees, are computed by specific algorithms without the need to formulate prior hypotheses or baseline models, the researcher’s intervention in the analysis is minimal (Witten and Frank, 2002).

### 1.1 Problem statement

**Figure 1:**
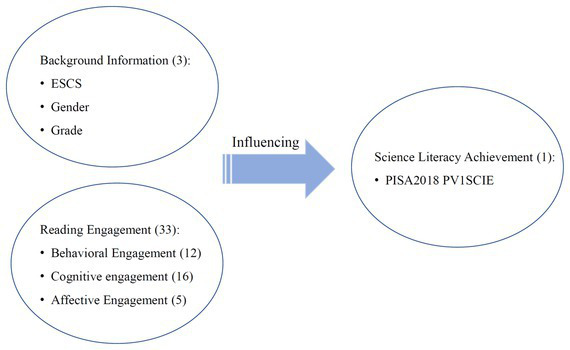
The conceptual framework of reading engagement and scientific literacy achievement.

In this study, we will take the student characteristic that has the greatest correlation with reading literacy scores, reading engagement, as the entry point to examine the relationship between students’ reading engagement and scientific literacy in terms of reading frequency and reading diversity (behavioral engagement), reading interest (affective engagement), and reading strategies (cognitive engagement). Using the XGBoost model under the GBRT algorithm in the data mining technology, the background information (family socioeconomic and cultural status, gender, grade level), reading frequency, reading diversity, reading interest, and reading strategy related indicators from the student questionnaire data of four provinces and cities in China of PISA2018 were used as the input variables in total 36 variables, and the scores of the Science Literacy Assessment (SLA) were used as the outcome variables to build a decision tree integration model. The feature importance, SHAP value and other indicators in the algorithm were used to interpret the influence of reading input-related variables on scientific literacy and explore the interaction between reading input-related variables. The conceptual framework of the independent variables and the dependent variables is shown in Figure 1.

### 1.2 Research question

Benefiting from the comprehensiveness of the international assessment program PISA data, this study focused on reading engagement and use PISA2018 data to explore the impact of reading engagement on scientific literacy. There are two main research questions to explore:

1. Which type of engagement hold the critical component for influencing the scientific literacy performance?
2. How the performance of the XGBoost method and the interpretation based on SHAP value under the condition of plenty of independent variables?

### 1.3 Significance

The significance and value of this study is mainly reflected in the following three aspects:

Firstly, the theoretical significance, this study explores the impact of reading engagement on scientific literacy, and comprehensively examines the relationship between reading engagement and scientific literacy in China’s 15-year-old student population from the three dimensions of behavioral, affective, and cognitive engagement, which is the gap of the existing research, and this study expands the impact of reading engagement beyond the field of reading, and enriches the people’s understanding of reading engagement.

Secondly, on the practical level, reading engagement is intervenable and has strong plasticity, this study is based on the current situation of reading engagement in China’s current group of 15-year-old students, with the intention of discovering new perspectives on improving students’ scientific literacy, which can start from the key variables found in the model by combining the key variables with the impact pattern of the outcome variable, i.e., scientific literacy, to provide feasible suggestions for education and teaching, and on the basis of which experts in the field of education and teaching apply pedagogy-related theories and practical experience to improve education and teaching; on the other hand, this study provides schools and policy makers with research evidence on relevant policy measures, with a view to promoting the improvement of the level of scientific literacy of young people in China.

Finally, in terms of research methodology, considering a large number of independent variables, this study uses the XGboost algorithm under the GBRT series, which is more mature but has not yet been widely applied to educational data, which has been used in many studies in the fields of sociology and pedagogy, and the feasibility and applicability have been proved, and combined with the SHAP value to visualize the model results, and the interpretation of the results, this study can, to a certain extent, enrich the evidence of the feasibility of machine learning related algorithms in the analysis of educational assessment data, and provide methodological reference for subsequent research.

## 2 Materials and method

### 2.1 Measures of reading engagement

In 2000, the PISA program used students’ reading attitudes to reflect reading engagement, namely, students’ reading interest scale, which was the earliest examination of reading engagement in PISA. In the subsequent PISA programs, the framework for measuring reading engagement was expanded, and the points of investigation were increased to include students’ reading time and the types of reading materials, etc. The PISA program carried out in 2009 mainly examined reading literacy, and improved the framework for reading engagement in the questionnaire system of that year, and for the first time, it made an all-around assessment of reading engagement from the engagement in three aspects: behavioral, affective and cognitive, and pointed out that a high level of reading engagement implies a high level of reading motivation, which could be reflected in a series of affective and cognitive engagement. Engagement implies a high level of reading motivation, which could be reflected in a series of emotional and behavioral characteristics, including showing interest in reading, enjoying reading, choosing reading content purposefully, and actively participating in diverse reading (OECD, 2010). In the current framework, “reading habits” is used to denote behavioral and affective engagement, and “reading strategies” is used to denote cognitive engagement, and PISA2018 has been adapted to examine, at the level of behavioral engagement, the followingPISA2018 adjusts on this basis, examining “reading frequency and variety” for behavioral engagement, “reading interest” for affective engagement, and “reading strategies” for cognitive engagement (OECD, 2019). In recent years, researchers on issues related to reading engagement have focused on the PISA scale, and most of the self-administered scales have built their assessment frameworks on the basis of the PISA reading engagement framework, which has performed well in all aspects of the scale. So far, PISA’s framework for measuring reading engagement has matured. Based on this, the reading engagement in this study adopts the indicator framework of PISA2018, which covers behavioral, affective, and cognitive engagement, specifically, behavioral engagement can be reflected in the frequency and variety of reading, affective engagement can be expressed as reading interests, and cognitive engagement can be reading strategies.

In this study, we took reading engagement, the student characteristic that has the greatest correlation with reading literacy achievement, as the entry point, and examine the relationship between students’ reading engagement and scientific literacy in terms of reading frequency and reading diversity (behavioral engagement), reading interest (affective engagement), and reading strategies (cognitive engagement). Using the XGBoost model under the GBRT algorithm in the data mining technology, the background information (family socioeconomic and cultural status, gender, grade level), reading frequency, reading diversity, reading interest, and reading strategy related indicators from the student questionnaire data of four provinces and cities in China of PISA2018 were used as the engagement variables, and the scores of the Science Literacy Assessment (SLA) were used as the outcome variables to build a decision tree integration model. The feature importance, SHAP value and other indicators in the algorithm were used to interpret the influence of reading engagement-related variables on scientific literacy and explore the interaction between reading engagement-related variables.

#### 2.1.1 Data

The sample data were obtained from the official public documents of PISA2018, including the literacy test and the background questionnaire. There were four cities in China (Beijing, Shanghai, Jiangsu, and Zhejiang) took PISA2018, and we have gotten the full sample data of these four cities for analyzing from the official PISA website regarding the representation of China.

The Chinese government agreed with the OECD to choose these four provinces and cities because they are at the forefront of China’s economic development and educational reform, have a basis for comparison with developed countries, have a high level of informationization in education, and have the conditions to participate in the test (the students had to answer the questions on a computer) (PISA 2018 Test Results Officially Released, 2019).

PISA2018 implemented a two-stage sampling, with a total of 12,058 students from 361 schools within mainland China participating in the assessment, and by assigning weights to the data for the calculations, the 12,058 data represented the overall 992,302 mainland Chinese students aged around 15 years old (81% are 15 years old).

Overall data: Of the 12,058 samples, 5,775 were female students (47.9%) and 6,283 were male students (52.1%). Participating students were mainly in the 9th and 10th grades, totaling 87%.

#### 2.1.2 Variable

The variables in this study were derived from the literacy test and background questionnaire in the PISA2018 program. The literacy test uses a format that includes open-ended, multiple-choice questions, etc., to sample students, and for the student response data according to Item Response Theory (IRT) technology, calculating each student’s ability or performance in the assessment area, including scientific literacy, reading literacy. The questionnaires were divided into principals, teachers, and students, and the corresponding questionnaires were used. The questionnaires mainly consisted of survey questions and scales to collect information about the participants’ family situation, learning status, and other information, which was rich in information.

The background information, reading engagement related variables selected in this study were derived from the student questionnaire. Scale scores in the PISA 2018 student questionnaire were calculated using IRT and parameter estimation was done using Weighted Likelihood Estimation (WLE) (OECD, 2019). There are 36 independent variables (including 3 background information variables, and 33 dependent variables), and 1 dependent variable (Science Literacy Achievement).

##### 2.1.2.1 Independent/input variables

###### 2.1.2.1.1 Background information

- Household Socioeconomic and Cultural Status (ESCS): 9 questions synthesized from three indicators: household possessions, parents’ highest occupational status, and parents’ highest level of education.
- Gender.
- Grade: In the PISA test, the grade value is a relative indicator; according to the Chinese school system, a 15-year-old student should be enrolled in the 10th grade, which is used as a criterion and is recorded as 0, if he/she is enrolled in the 11th grade it is recorded as 1, if he/she is enrolled in the 9th grade it is recorded as −1, and so on.

###### 2.1.2.1.2 Reading engagement

- Behavioral Engagement: is portrayed by two indicators, Frequency of Reading and Diversity of Reading, which focuses on how well students read a variety of topic types of texts in their daily lives, with the specific choices being “ST167 How often do you read the following types of reading materials because of personal preference?” “ST175 How much time do you usually spend reading for pleasure?” “ST176 Do you regularly engage in the following reading activities?” These are three sets of questions. Of these, ST167 contained 5 items and ST176 contained 6 items, yielding a total of 12 variables.
- Cognitive engagement: portrayed by reading cognitive strategies, which were examined in three dimensions, namely comprehension (ST164), summarization (ST165), and evaluation (ST166), requiring the respondents to rate the usefulness of the strategies shown in the options according to the question context, with higher scores on the variables indicating that the student has a better grasp of this type of reading strategy. A total of 16 items measure cognitive reading strategies.
  - The “Comprehension” question context was “Requires understanding and remembering information in a text” and consisted of six items, such as “Discussing the content with others after reading.”
  - The question context for assessing “summarization” was “Summarize a long and complex text about fluctuations in the water level of a lake in Africa,” with 5 items, e.g., “Try to copy as many sentences as possible as accurately as possible from the original text.”
  - The context in which “assessment” was measured was the one about the “email with information about winning a lottery from an unknown source,” with 5 items, e.g., “Respond to the email asking for more information about the functioning cell phone.”
- Affective Engagement: portrayed by Interest in Reading (ST160), which focuses on students’ attitudes toward reading and asks “To what extent do you agree or disagree with the following statements about reading?” and a total of 5 items.

See Supplementary material for more details on the above questions.

##### 2.1.2.2 Dependent variable (output)

**Figure 2:**
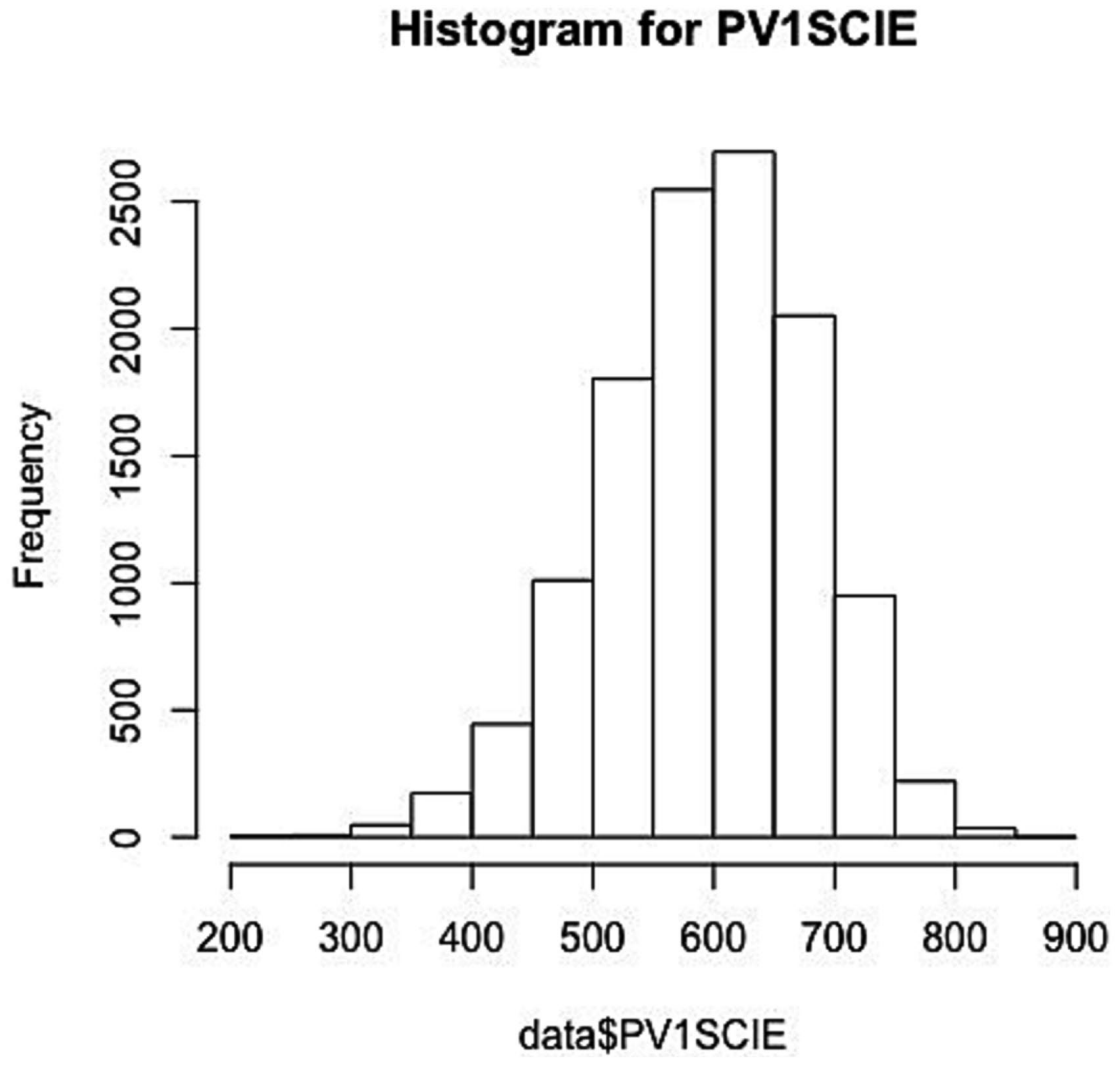
The frequency distribution of output.

Science Literacy Achievement (continuous variable). For students’ scientific literacy achievement, PISA is characterized by PV (Plausible Value) values in 10 groups. PV values can be referred to as likelihood values, which represent the range of abilities that a student may possess. Modern measurement theory suggests that it is more scientifically sound to consider the probability distribution of a student’s ability, and that in the past a simple estimate of ability was unreliable in representing a student’s ability. In view of the fact that PV values can provide unbiased estimation of the overall parameters, only one set of PV values was selected for analysis in this study. Its frequency distribution is shown in Figure 2, and the distribution is roughly normal.

### 2.2 Method

Traditional mathematical and statistical analysis methods (e.g., regression modeling, structural equation modeling) are model thinking, which is to construct a model and then embed the data for fitting, and the best fitting model is derived by comparing the fitting of different models to explain the data; while machine learning is typical data thinking, thanks to the development of computer computing power, which has been developing rapidly in many fields in recent years, such as biology, medicine, economy, etc., and the overall idea is to let the computer form a model through the iteration of the algorithm, which has a clear advantage in the case of more variables, complex and unclear data structure, and can dig out more data features and discover potential data patterns. For example, a researcher used machine learning algorithms in the health aspect to predict mental illness (Nayan et al., 2022). The researcher compared six machine learning algorithms, including namely logistic regression, random forest (RF), support vector machine (SVM), linear discriminate analysis, K-nearest neighbors, and Naïve Bayes, and ultimately recommended that RF and SVM classification algorithms are more moderated in predicting college students’ mental health status, as well as being of core interest in the future. Of course, in data analysis, completely theory-driven or completely data-driven cannot be called a good analysis method. In different stages of data analysis, both theory and data itself should be paid attention to. In recent years, with the development of large-scale international education assessment, education data has gradually become big data, and large-scale assessments such as PISA and TIMSS contain a large amount of data: cognitive and non-cognitive factors at the student level, the teaching situation and job identity at the teacher level, as well as a series of variables at the school level and the family level, and data from the assessment process. These data contain a great deal of information, and the use of machine learning can effectively address the limitations of traditional mathematical and scientific methods.

#### 2.2.1 XGBoost algorithm

The XGBoost algorithm was proposed by Chen and Guestrin (2016) and is known as eXtreme Gradient Boosting. The model is built in the framework of gradient boosting model and developed from GBRT (Gradient Boosted Regression Trees, GBRT), which is the GBRT algorithm’s engineering implementation.

GBRT is a tree ensemble model for prediction of dataset A. Given a dataset A, containing *N* samples and M features, formed by K accumulative functions with the following formula Equation (1):

(1) $$\hat{y_{i}}=\emptyset \left(x_{i}\right)=\overset{K}{\underset{k=1}{\sum }}f_{k}\left(x_{i}\right)，f_{k}\in F，\left(i=1,2,3,\ldots ,N\right)$$

where $F=\left\{f\left(x\right)=w_{q\left(x\right)}\right\}\left(q:R^{m}\to T,w\in R^{T}\right)$ denotes the space of regression trees, where each $f\left(x\right)$ represents a separate tree as a function of the structure q and the weight w of a node, and T denotes the depth of the tree. The structure q itself is also a function, and $q\left(x\right)$ denotes the assignment of a sample x to a node of the tree.

The significance of the model objective function is that it measures how well the model fits the training data, which can also be referred to as the loss function, and evaluates the difference between the predicted value obtained by applying the model and the true value, as defined below Equation (2):

(2) $$L\left(\emptyset \right)=\underset{i}{\sum }l\left(y_{i},\hat{y_{i}}\right)+\mathrm{constant}$$

A smaller loss function indicates a better fit.

GBRT as an integrated algorithm, each new tree can be viewed as adding a new iteration round, the goal of the new iteration is to minimize the residuals under the previous model of the previous iteration, the formula is: expressed as followed Equation (3):

(3) $$F_{k}\left(x\right)=F_{k-1}\left(x\right)+\gamma_{k}f_{k}\left(x\right)$$

where $f_{k}\left(x\right)$ denotes the $k_{th}$ tree and $\gamma_{k}$ can be interpreted as the weight of this $k_{th}$ tree.

The loss function is not only have one form, Friedman (2001) proposes to replace the approximation of the loss in the current iteration with the negative gradient of the loss function, which is also the core of the GBRT algorithm. The gradient is the directional derivative of the function at a point where the function changes the fastest, and using this method allows the number of iterations to be greatly reduced. For the $k_{th}$ iteration, the negative gradient on $x_{i}$ is as followed Equation (4):

(4) $$Z_{m}\left(x_{i}\right)=-{\left[\frac{\partial L\left(y_{i},F\left(x_{i}\right)\right)}{\partial F\left(x_{i}\right)}\right]}_{F\left(x\right)=F_{k-1}\left(x\right)}$$

$f_{k}\left(x\right)$ can be generated based on $\left(x_{i},Z_{ik}\;\right)\left(i=1,2,3,\ldots ,n\right)$ .

XGBoost essentially predicts the dataset as GBRT does, and the tree ensemble model is the same as Equation 1. A major difference lies in the model objective function, which is defined for XGBoost as followed Equation (5):

(5) $$L\left(\emptyset \right)=\underset{i}{\sum }l\left(y_{i},\hat{y_{i}}\right)+\underset{k}{\sum }\Omega \left(f_{k}\right)+\mathrm{constant}$$

where $\Omega f_{k}=\gamma T+\frac{1}{2}\lambda w^{2}$ , T denotes the number of nodes and w denotes the node score.

$\underset{i}{\sum }l\left({\hat{y\;}}_{i},y_{i}\right)$ is the loss function part, the most commonly used loss function is the mean squared error $L\left(\theta \right)=\underset{i}{\sum }{\left(y_{i}-{\hat{y}}_{i}\right)}^{2}$ , and there are other loss functions, such as the logisitic function: $L\left(\theta \right)=\underset{i}{\sum }\left[y_{i}\ln\left(1+e^{-{\hat{y}}_{i}}\right)+\left(1-y_{i}\right)\ln\left(1+e^{{\hat{y}}_{i}}\right)\right]$ ; compared with GBRT, the XGBoost toolkit allows users to define their own loss function. $\underset{k}{\sum }\Omega \left(f_{k}\right)$ is the regularization term, which can effectively control the complexity of the model.

Compared with GBRT, the XGBoost model iteration process takes the loss function one step further and uses a second-order Taylor expansion, which can accelerate the model convergence. For the $k_{th}$ iteration, the objective is to find $f_{k}\left(x\right)$ such that the following equation is minimized as followed Equation (6):

(6) $$\begin{array}{l} L_{k}\left(\emptyset \right)=\underset{i}{\sum }\left[l\left(y_{i},{\hat{y_{i}}}^{k-1}\right)+g_{i}f_{k}\left(x_{i}\right)+\frac{1}{2}h_{i}{f_{k}}^{2}\left(x_{i}\right)\right]\\ \;+\Omega \left(f_{k}\right)+\mathrm{constant} \end{array}$$

where $g_{i}=\partial_{{\hat{y_{i}}}^{\left(k-1\right)}}l\left(y_{i},{\hat{y_{i}}}^{k-1}\right)$ , $h_{i}={\partial_{{\hat{y_{i}}}^{\left(k-1\right)}}}^{2}l\left(y_{i},{\hat{y_{i}}}^{k-1}\right)$ (Chen and Guestrin, 2016).

The XGBoost algorithm has been widely used in various big data competitions in the past few years since it was proposed, obtaining remarkable results, and the robustness of the algorithm has been verified. Compared with other integrated algorithms, the XGBoost algorithm has the following advantages: (1) Effective avoidance of overfitting. The XGBoost algorithm could incorporate a regularization term in the objective function. (2) Highly informative to use. The model boosting phase requires a higher loss function, i.e., second-order derivable, and using both first-order derivatives and second-order derivatives could result in a greater amount of information and a more accurate loss calculation. (3) Allows for the presence of missing values in the training set. The model takes into account the sparse values of the training data, and can define the missing values or specify the branching direction for a particular value, which significantly improves the efficiency. (4) Supports multi-threaded operation. When the amount of data is relatively large can maximize the use of disk.

This study used the software R4.0.1 for pre-data cleaning and variable relationship analysis, and the tool used in the modeling and analysis stage is Python 3.6.13, mainly using the XGBoost (Chen and Guestrin, 2016) modeling algorithm, the core algorithm package is xgboost.

#### 2.2.2 Tuning parameters

In general, supervised machine learning requires three phases: training, tuning and testing. In the training phase, the model learns how to map each engagement value to an observation, a process similar to knowledge extraction by the human brain using a form of inductive reasoning. The tuning phase is a very critical part, the model could be different under different parameter settings, and the researcher needs to calibrate the parameters to achieve the best performance of the model. The important parameters in the XGBoost model are shown in Table 1, which are divided into three major categories, one is the general parameter category: mainly for macro function control; the second is the boosting parameter category: it is used to control the tree boosting in each step, the boosting parameter can generally control the model calculation; three is the learning target parameter class: mainly to define the target task, such as whether it is a regression or classification problem, if it is a classification problem furthermore, whether it is a binary classification problem or a multiclassification problem, which is set in the target parameter.

**Table 1:** XGBoost important parameters.

General parameters	
booster	Basic structure, optional gbtree/gblinear/dart, default value gbtree.
verbosity	Information output degree, 0–3, default value 1.
nthread	Number of parallel threads. The default value −1 indicates the maximum thread parallelism.

Parameters for tree booste	
eta/learning-rate	Update the shrink step used. The default value is 0.3 and the value ranges from 0 to 1.
gamma/min_split_loss	The minimum loss function required to form a tree node. The default value is 0.
max_depth	The maximum depth of the tree, the default is 6.
min_child_weight	Weight of samples within a node. The default value is 1.
subsample	Proportion of randomly sampled training samples per tree.
sampling_method	Sampling method.
colsample_bytree	The proportion of columns sampled during tree generation.
lambda	L2 regularization parameter that controls the complexity weight value of the model
tree_method	Tree construction algorithm.

Learning task parameters	
objective	Learning tasks and learning objectives. The default value is reg:linear.
base_score	Specify a global offset for the sample prediction.
eval_metric	Specified evaluation index.
seed	Specify the random number of seeds and the results of the random results.
num_boost_round	Specifies maximum iteration times.
early_stopping_rounds	The specified iteration is not optimized and stops training.

The training, tuning and testing phases should use different data to avoid the risk of overestimating the model performance, which can be achieved by using k-fold cross-validation (Hastie et al., 2001), which randomly divides the available data (i.e., the set of solved cases) into k subsets, and the model is first trained on the k−1 subsets and then tested on the remaining subsets. This process is repeated k times, averaging the model performance results of these k times to obtain more stable estimates (Calanna et al., 2020).

#### 2.2.3 Model building

After data cleaning the model can be constructed: scientific literacy as the outcome indicator and 36 raw variables of key examination factors are used as engagement variables.

The target dataset was first divided into training and test sets. The random seed is set, and the studied dataset is randomly divided into three parts, two of which are training set for training the model and applying cross-validation and grid search to find the optimal parameter combinations, and the remaining one is used as a test set for the validation of the model’s generalization ability.

The model building can be divided into three main steps, the first step is to establish the parameter initialization model (Model 1), each parameter adopts the default value, and it is used as the baseline model for the comparison of the model after parameter tuning; the second step is to use the methods of cross-validation and GridSearchCV to adjust the key parameters in the model to find the optimal parameter combinations, i.e., the parameter tuning, which is already introduced in the previous part of the research design. The parameters in the XGBoost model algorithm can be divided into three major categories, i.e., general, enhancement and learning target, and the parameters that have a greater impact on the model performance are concentrated in the enhancement and learning target parameters; the third step is to form an optimal model based on the optimal parameters obtained in the second step, and complete the final model building (Model 2).

In the XGBoost model, the number of iterations plays a key role in the learning target parameters and is usually the first parameter to be adjusted. For any model, the higher the number of iterations, the better it fits or even overfits that data set.

**Figure 3:**
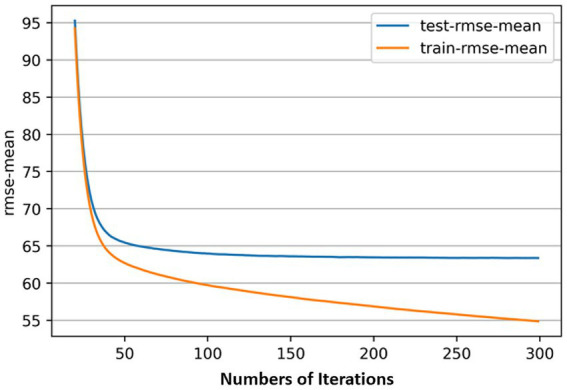
Relationship between the number of iterations and RMSE-mean of model.

In this study, the search for the optimal number of iterations is performed using the cross-validation method with the root mean square error (RMSE) as the model fit metric. There are two main parameters to control the iteration range, namely num_boost_round and early_stopping_rounds, the former determines the maximum number of iterations of the model in training, and the latter sets the rule of early termination of iteration, meaning that iteration is automatically stopped if the evaluation metrics have not been reduced after a number of rounds in the process of reaching the maximum number of iterations. By setting num_boost_round to 500 and early_stopping_rounds to 100, the model automatically stops after 282 iterations. Make a graph to see the trend of the RMSE mean change from 0 iterations to 300 iterations, as shown in Figure 3, it can be found that when the number of iterations exceeds about 100, as the model’s fit to the training set increases, the model’s fit to the test set could remain within a certain level, and could no longer have a significant improvement.

Specifically look at the lifting parameters: eta/learning-rate controls the weight reduction of each learning, to provide more learning space for the later model, in this study, assigned values 0.1, 0.2, 0.25, 0.3, 0.4 for adjustment; parameter gamma/min_split_loss indicates that leaf node splitting requires the minimum amount of 0.5, 0.7, 0.8, 0.9, 1 for tuning.

Boosting the parameter tuning settings yields 2,400 combinations using 5-fold cross-validation, which ultimately ran 12,000 times. The final optimal parameter combinations obtained were a learning rate of 0.1, a minimum split loss of 0.05, a maximum tree depth of 3, a minimum number of branching samples of 3, and a random sampling ratio of 0.8 per tree.

The parameter settings for Model 1, tuning parameter selection and Model 2 are shown in Table 2.

**Table 2:** XGBoost parameter setting.

Parameter	Default value model 1	Tuning parameters	Optimal combination model 2
**General parameters**			
booster	gbtree	gbtree	gbtree
verbosity	2	2	2
nthread	−1	−1	−1
**Parameters for tree Boostr**			
eta/learning-rate	0.3	[0.1, 0.2, 0.25, 0.3, 0.4]	0.1
gamma/min_split_loss	0	[0.05, 0.1, 0.2, 0.3]	0.05
max_depth	3	[2, 3, 4, 5, 6, 7, 8, 9]	3
min_child_weight	1	[1, 2, 3, 4,5,6]	3
subsample	1	[0.3, 0.5, 0.7, 0.8, 0.9, 1]	0.8
sampling_method	uniform	/	uniform
colsample_bytree	1	/	1
lambda	0	/	0
tree_method	auto	/	auto
**Learning task parameters**			
objective	reg:squarederror	reg:squarederror	reg:squarederror
base_score	0.5	0.5	0.5
eval_metric	rmse	rmse	rmse
seed	10	10	10
num_boost_round	100	500	282
early_stopping_rounds	0	100	0

#### 2.2.4 Model evaluation and interpretability

In the xgboost algorithm toolkit, the evaluation function, eval_metric, is commonly used as an indicator for evaluating the excellence of a model, and seven different calculation methods are provided, namely, Root Mean Squared Error (RMSE), Mean Absolute Error (MAE), Negative Log Likelihood Function (LOGLOSS), Binary Classification Error Rate (MERROR), Multi-classification Error Rate (MERROR), Multi-classification LOGIT Loss Function, and Area under the Curve (AUC), which can be chosen by the researcher according to the research needs and data characteristics, and can also be customized to evaluate the function, which is generally based on the distance between the predicted value and the true value for consideration.

For the interpretability of the model results, researchers advocate model-agnostic interpretable methods, which are intended to get rid of the model limitations and analyze the interpretable part of the model. One of the better fit with XGBoost is SHAP value (Shapley Additive Explanations value), proposed by Lundberg and Lee (2017) inspired by cooperative game theory, which is an additive interpretation method that is widely applicable to explaining a variety of models, including XGboost models. For any model, each sample could produce a predictive value based on a specific model, in the more widely used linear regression model, the model user can view the regression coefficients to intuitively understand the impact of different components on the results; while in machine learning such as the decision tree integration model, in the face of a large number of different decision trees, the researcher often gets the results but is unaware of the specific process of their formation, the SHAP value could provide the values assigned to each feature in a single sample, providing the researcher with the perspective to observe how the variables/features affect the results.

Similar to the summation method for linear models, assuming that the model base score, i.e., the mean value of the target variable across all samples, is $y_{\mathrm{base}}$ , the $i_{th}$ sample is $x_{i}$ , the $j_{th}$ feature of the $i_{th}$ sample is $x_{i,j}$ , and the SHAP value of that feature is $f\left(x_{i,j}\right)$ , then the model’s predicted value for that sample is $y_{i}$ , which is calculated by the following formula Equation (7):

(7) $$y_{i}=y_{\mathrm{base}}+f\left(x_{i,1}\right)+f\left(x_{i,2}\right)+\ldots +f\left(x_{i,j}\right)$$

When $f\left(x_{i,1}\right)>0$ , it means that the feature enhances the prediction value, i.e., positive effect; $f\left(x_{i,1}\right)<0$ means that the feature makes the prediction value lower, negative effect.

## 3 Results

### 3.1 Model fit

**Figure 4:**
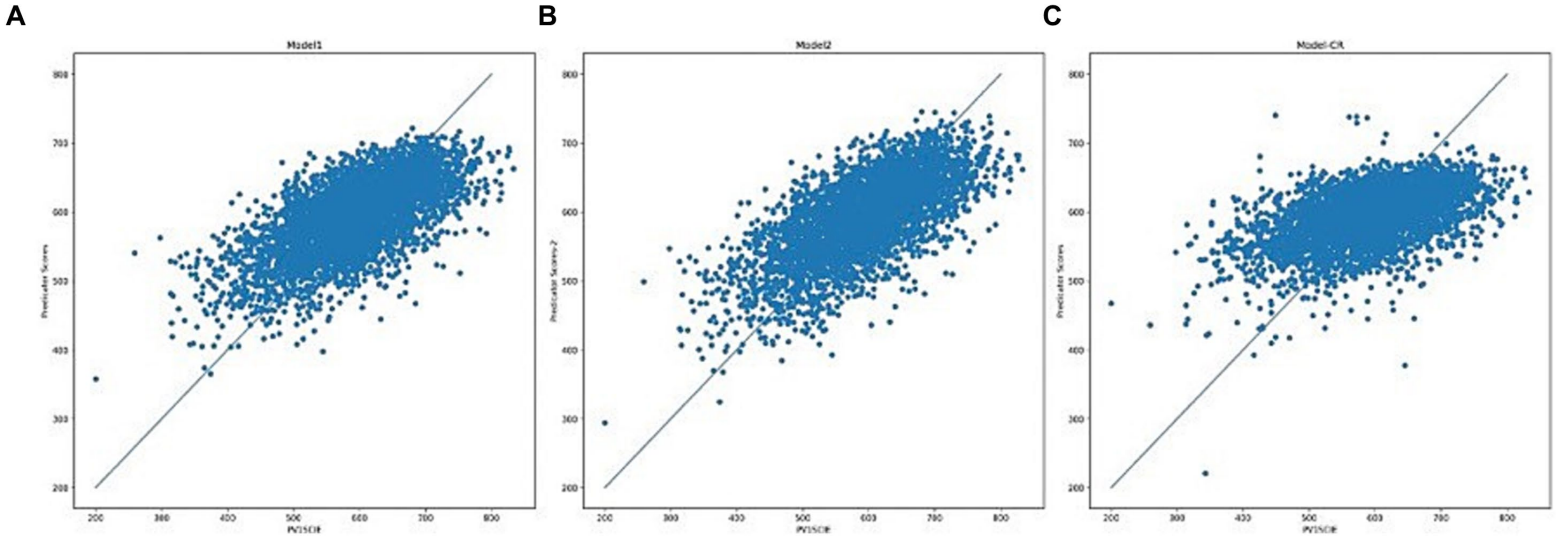
Scatter plot of test set outputs **(A)** Model 1, **(B)** Model 2, and **(C)** Regression model.

Model 1, the default model, $r^{2}$ scored 0.504 on the training set and $r^{2}$ scored 0.443 on the test set; Model 2, the best model under the conditions of this study, obtained after tuning the parameters, $r^{2}$ scored 0.564 on the training set and $r^{2}$ scored 0.447 on the test set, which is the model that could be used for the subsequent analyses of this study. The predictions of the results of the initial model 1 and the tuned Model 2 for the test set are shown in Figures 4A,B, exhibiting similar distribution patterns. The model metrics for Model 1 and Model 2 on the training set are shown in Table 3, and the model metrics on the test set are shown in Table 4. The tuned model shows a more significant improvement in the fit on the training set compared to the initial model, and the test set also shows a slight improvement in some of the model metrics, indicating that the model has not yet been overfitted, and it performs well on this type of dataset.

**Table 3:** Model fitting index – training set.

Index	Model 1 (model_default)	Model 2 (model_tuned)	Regression model
MSE	3264.499	2626.409	5677.856
RMSE	57.136	51.249	75.352
MAE	45.103	40.230	59.458
R2	0.504	0.564	0.236

**Table 4:** Model fitting index – test set.

Index	Model 1 (model_default)	Model 2 (model_tuned)	Regression model
MSE	3989.779	4284.802	5497.426
RMSE	63.165	63.459	74.145
MAE	49.741	50.118	58.541
R2	0.443	0.447	0.243

In order to better evaluate the XGBoost model under the task of this study, the same training set and test set were used to build the regression model, and its prediction of the test set is shown in Figure 4C, and the indicators are shown in Tables 3 4, which show that the XGBoost algorithm has a clear advantage, whether it is the tuned model or the default model.

### 3.2 Interpretability

#### 3.2.1 Global interpretation

##### 3.2.1.1 Feature importance

Interpretation of XGBoost results using SHAP values to measure the importance of features. This is a more general perspective that considers the extent to which individual features contribute to the model’s prediction score values.

**Figure 5:**
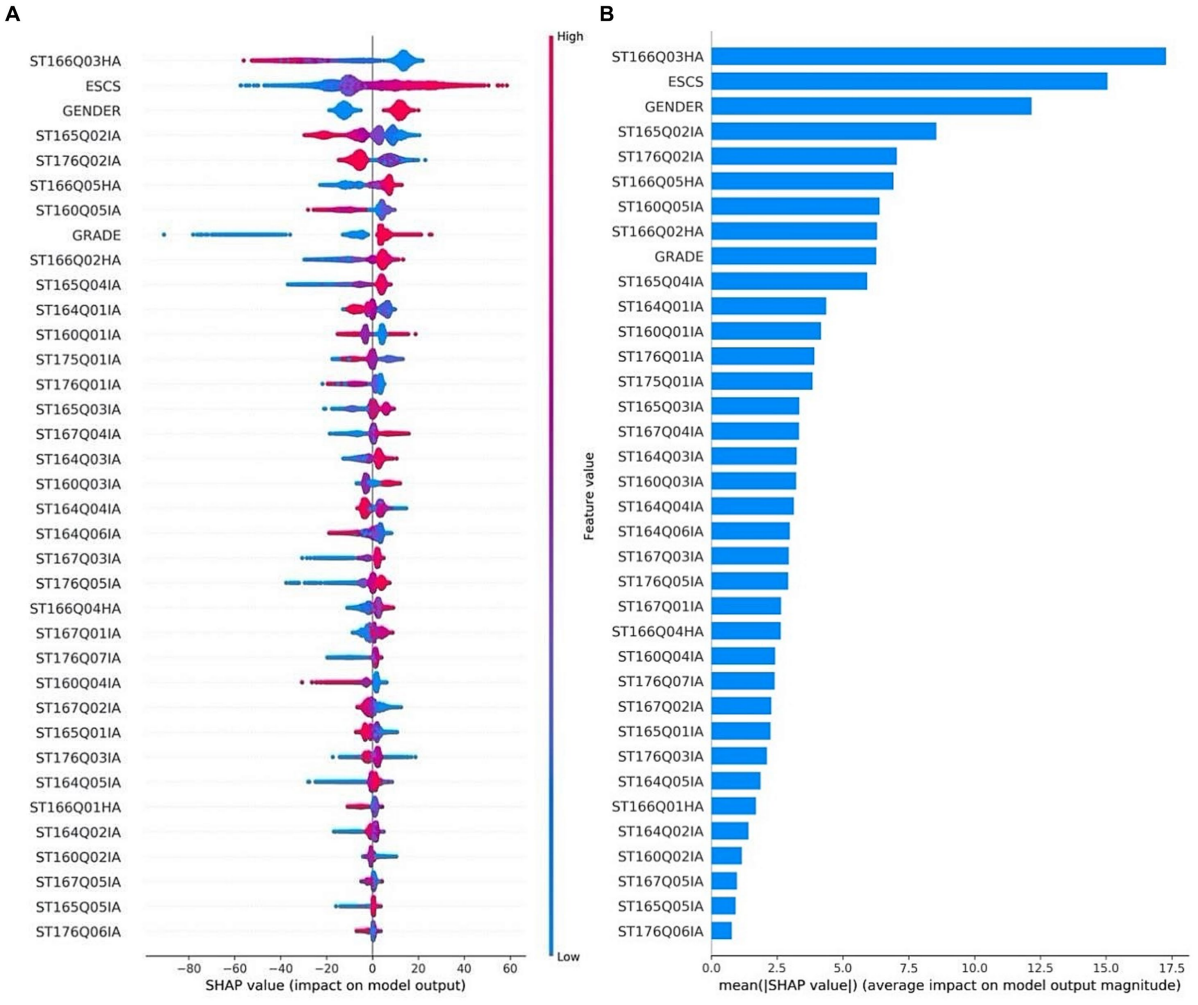
SHAP value of each feature **(A)** distribution and **(B)** average SHAP value ranking.

The distribution of SHAP values for each feature is shown in Figure 5A, the ranking is based on the average of the SHAP values, the average is shown in Figure 5B, the size of the average reflects the contribution of the feature in the model prediction, which can be used as an indicator of the importance of the feature, the highest average SHAP value is ST166Q03, the lowest is ST176Q06, the difference between the two is about 17 points. Each row in Figure 5A represents a feature with the SHAP value in the horizontal coordinate. A dot represents a sample, and blue to red indicates that the value of the feature itself is increasing. The distribution of SHAP values for the ST166Q03 “Click on the link as soon as possible to fill in the profile” strategy is more dispersed, which has a larger impact on the sample, and is consistent with the previous analysis, which shows that the SHAP values for this strategy are negative when the strategy is evaluated more highly, which means that the final scores could move in a negative direction. The global SHAP value of ESCS is more widely distributed and is a relatively important feature, the larger the value the larger its SHAP value, which is positively correlated with the prediction results. Gender is more favorable for boys than for girls. Grade level also significantly affects science literacy scores, but the main influence is on the small grade level group, with small grades predicting fewer scores and a gap of about 70 from the normative grade level students.

##### 3.2.1.2 Interaction

SHAP value analysis can be viewed as a model disassembly process. In the XGBoost model, each feature contributes to the prediction to a different degree, and together they change the prediction from the mean to the final value. The interaction of the variables can be observed by looking at the change in SHAP value when two variables work together.

**Figure 6:**
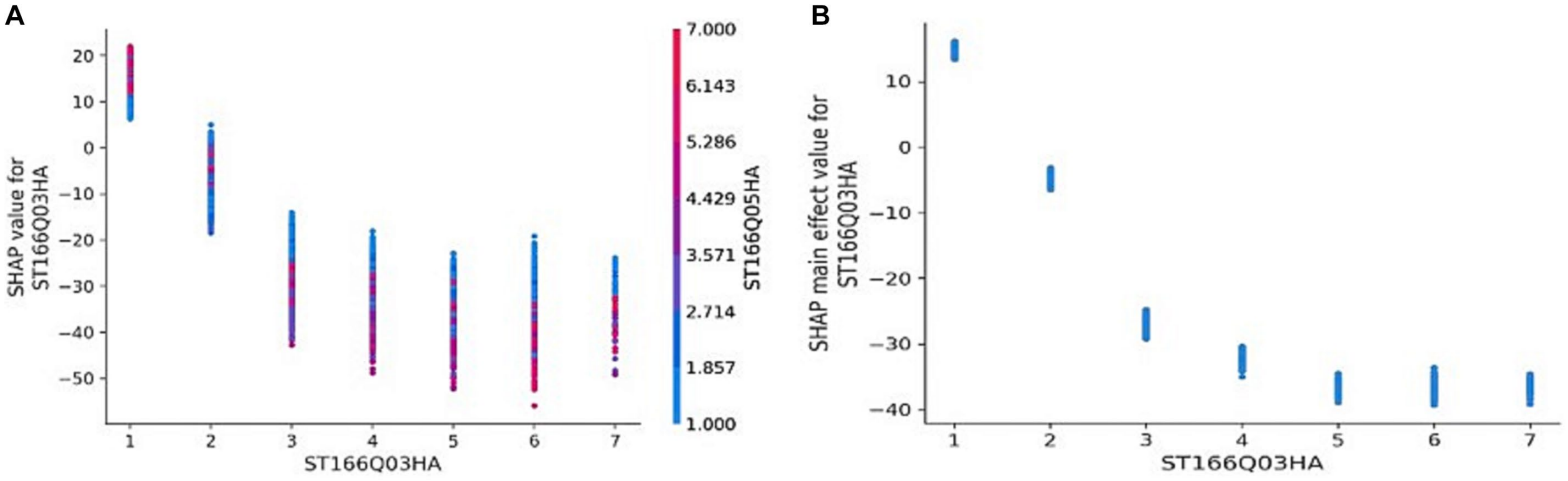
Interaction – ST166Q03.

Interaction analysis is performed on the top five features in terms of importance. The program was set to automatically find the variables with which it had the most significant interactions and visualize them. The first one is ST166Q03, and among the remaining 35 feature variables, the interaction between ST166Q05 and it is the most obvious, which is also the variable with the second highest importance of the feature, as shown in Figure 6A: under the influence of ST166Q05, when ST166Q03 is equal to 1, the SHAP value fluctuates between 5 and 22 points, and the higher the value of ST166Q05, the higher the value of ST166Q03, the greater the SHAP of ST166Q03, i.e., the higher the model’s prediction of students’ scientific literacy scores when they evaluate both strategies “correctly.” When ST166Q03 is greater than or equal to 2, the SHAP values are off the negative axis, and the fluctuation range is influenced by ST166Q05, which is roughly a 30-point fluctuation interval, and the higher the ST166Q03, the smaller the SHAP value is, compared with the SHAP value of ST166Q03 without the effect of ST166Q05 as presented in Figure 6B, the fluctuation interval is obviously increased, and there is an overlapping area.

#### 3.2.2 Local interpretation

In addition to global interpretation, SHAP analysis can also provide local interpretation of the model by specifying a sample or samples for interpretation.

For each sample, the role of each characteristic variable on this sample could be specified with a one-to-one correspondence of SHAP values, and the SHAP values of each characteristic could be accumulated and then added to the baseline value of the model to get the predicted value of the model for the sample, when the SHAP value is positive, the model predicts that the predicted score of scientific literacy of the sample shifted in the positive direction from the baseline value to the numerical axis, and vice versa. Move in the negative direction.

**Figure 7:**
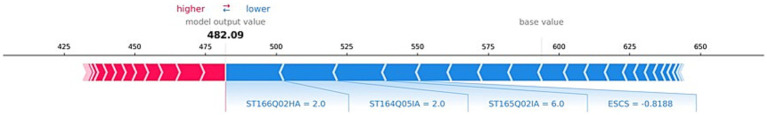
Visualization of SHAP values for the features of sample No. 30.

**Figure 8:**
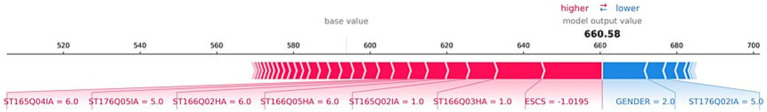
Visualization of SHAP values for the features of sample No. 6860.

Taking No. 30 and No. 6860 students as an example, by calculating the mean value of the model’s prediction scores on the training set, the model baseline score is obtained as 593.82. Sample 30 final model score is 482.09 and sample 6,860 final model score is 660.58. The contribution of features to the predicted sample scores is visualized in Figures 7, 8. It can be seen that the four features that have a greater impact on the final score of Sample 30 are ST166Q02HA, ST164Q05IA, ST165Q02IA, and ESCS, which have a negative impact. And the features that have a greater impact on sample 6,860 are ESCS, ST166Q03HA, ST165Q02IA, and so on.

**Figure 9:**
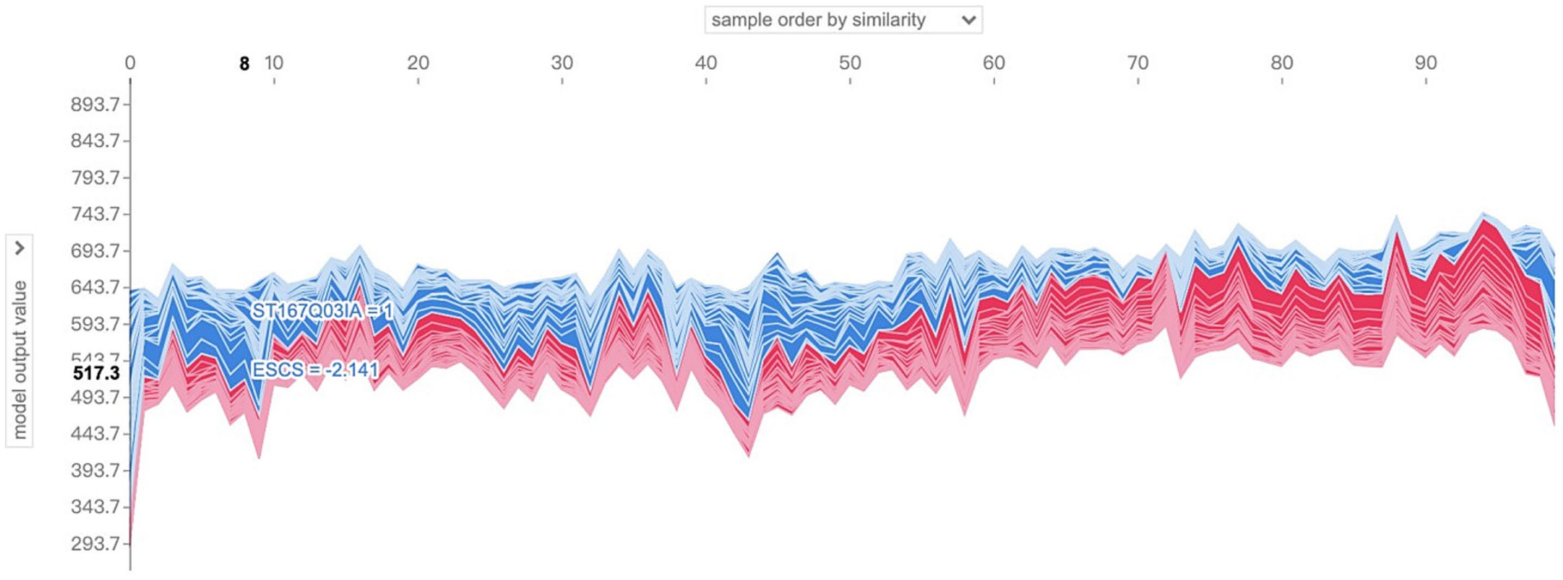
Visualization of SHAP values for the features of sample No. 100–No. 200.

Further, the situation of the characteristic SHAP values of several samples can be examined simultaneously. In the localized SHAP value analysis, it is allowed to limit the range of samples to be visualized, which can be interpreted as a collection of visual images of the characteristic SHAP values of individual samples, i.e., a combination of several Figures 7, 8, such as shown in Figure 9, for which the SHAP-related package provides an interactive image. Take the sample number 100 to 200 as an example, as shown in Figure 9, in the upper drop-down box, select the horizontal axis to present the content and logic of the “sample order by similarity” that is, to present the distribution of the SHAP value of the 100 samples, the blue color indicates the positive SHAP value, the red color indicates the negative SHAP value; The presentation is not in the order of the sample number, but has higher similarity samples together; vertical axis through the left drop-down box to select the “model output value” that is, the output results; when moving the cursor in the image area, you can instantly view the predictive scores of a sample (in the vertical axis of the display of values) and When you move the cursor in the image area, you can instantly view the prediction score of a sample (the value is shown in the vertical axis and bolded) and its SHAP value of the larger features and their values, and the tip of the sample number also appeared at the top of the operating interface. Analyzing the graph, by moving the cursor, it is found that the majority of the samples presented in the right half of the image have model prediction scores above 600, while the left half is below 600, which intuitively seems to be due to the fact that the samples in the left half of the image have more negative SHAP values that have a larger impact, while the right half of the image has more positive SHAP values for the features.

**Figure 10:**
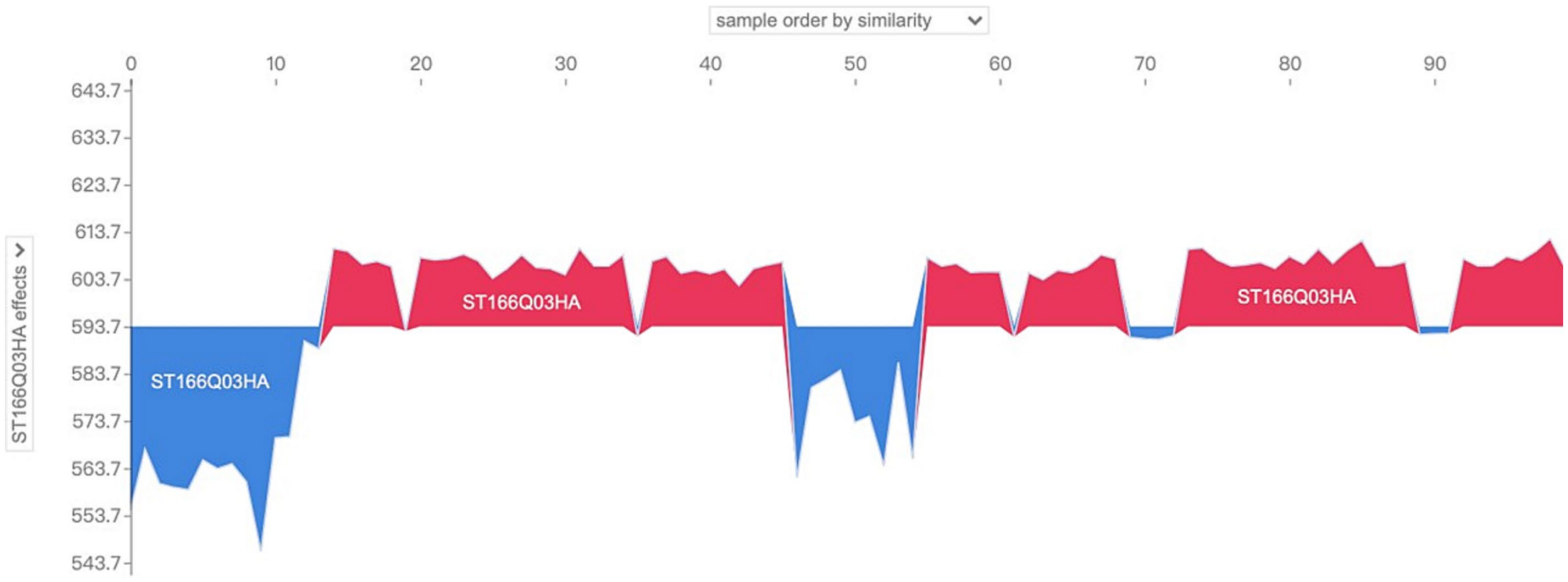
Visualization of SHAP values for the feature ST166Q03 of sample No. 100–No. 200.

**Figure 11:**
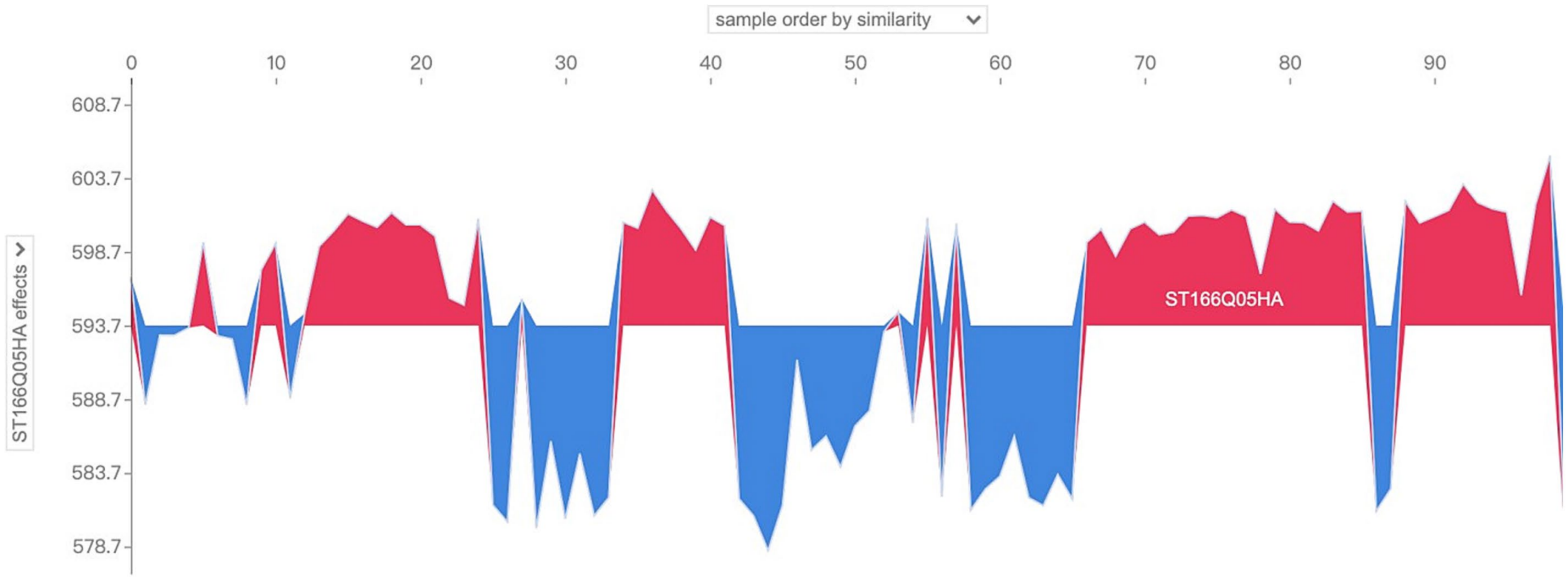
Visualization of SHAP values for the feature ST166Q05 of sample No. 100–No. 200.

**Figure 12:**
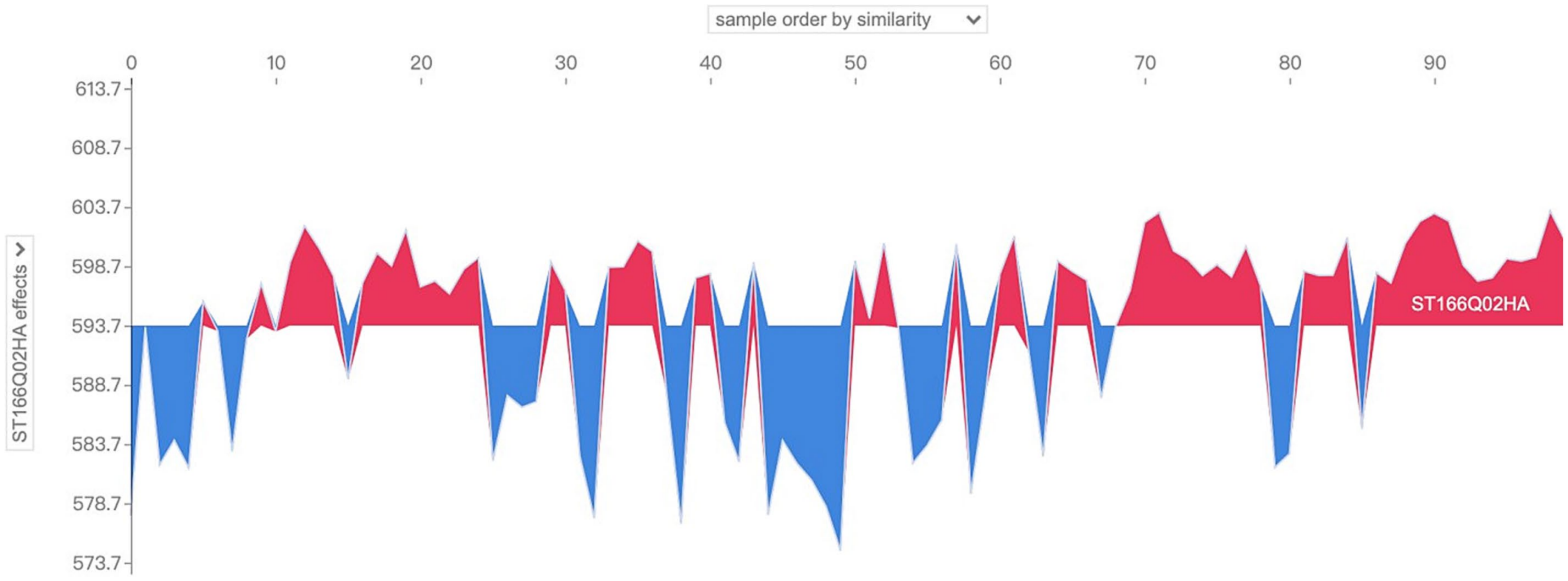
Visualization of SHAP values for the feature ST166Q02 of sample No. 100–No. 200.

So what are the main characterizing variables that contribute to the differences in the distribution patterns of SHAP values? The drop-down box on the left side provides all the characteristic variables of the model, which can be analyzed for characteristic effects to explore the distribution of SHAP values of different characteristic variables in the sample population. Taking the above sample as an example, change the drop-down box on the left to view the SHAP values of “ST166Q03,” “ST166Q05,” “ST166Q02,” “ST166Q03,” “ST166Q05,” and “ST166Q02,” which have a higher degree of characteristic importance in the previous analysis. SHAP value distribution, such as Figures 10–12, it can be found that the feature ST166Q03 produce negative SHAP value situation basically exists in the left half of the image of the samples; and comprehensive three images found that the SHAP value of the positive and negative patterns are a high degree of overlap, reflecting the existence of a certain degree of group commonality.

## 4 Conclusion

This study intends to investigate the relationship between reading engagement and science literacy assessment scores using a sample of 15-year-old Chinese middle school students. Using the XGboost algorithm in the machine learning model, the model was constructed by taking the variables related to reading engagement, including behavioral engagement, affective engagement, cognitive engagement, and demographic information as engagement, i.e., feature variables, and science literacy assessment scores as outcome variables.

Two basic conclusions were drawn from this study:

1. Reading cognitive engagement is the most critical component in this study’s model and plays an important role in the assessment of students’ scientific literacy.
2. The XGBoost model fits well with a large number of input variables, and the post-hoc interpretation method based on SHAP values, which is well visualized, has good prospects for application in the field of educational psychometrics.

## 5 Discussion

### 5.1 Cognitive engagement is the critical ingredient

Globally, among the three major components of reading engagement, i.e., cognitive engagement, affective engagement and behavioral engagement, there is a difference in the influence on the model’s predicted results, i.e., science literacy assessment scores. From the analysis of the SHAP values of the features, half of the top ten features in terms of their influence on the model are feature variables belonging to cognitive engagement; and for the top five features in terms of their importance, the variables that interacted with them more strongly were concentrated in the cognitive engagement-related variables.

In this study, cognitive engagement in reading refers to the high level of “involvement” of students’ mental resources in reading activities, which reflects students’ application of high-level cognitive strategies, monitoring and adjustment of the reading process. The beneficial effects of high levels of cognitive engagement in reading on students’ performance in science literacy assessment may come from two aspects: on the one hand, the testing process requires students to comprehend and analyze the test questions, and students who have mastered good metacognitive strategies for reading are able to understand the questions better and look for valuable clues to solve the questions. It has been shown (Chi, 2000; McCrudden et al., 2007) that deep cognitive engagement helps students to choose effective reading strategies, flexibly adopt the ways of guessing the meaning of words through the context, finding the central sentence and key words to interpret the meaning of the text, and the use of reading metacognitive strategies can speed up the process of information comprehension. The findings of a study (Kim et al., 2021) that showed a positive and significant effect of Model of Reading Engagement (MORE) on first grade students’ knowledge in science domains such as depth of vocabulary knowledge, listening comprehension, and argumentative writing are consistent with the findings of the present study. Some researchers (Sørvik and Mork, 2015) have also further proposed a framework in which texts play a role in science education. On the other hand, students with high cognitive engagement in reading would have more channels to acquire knowledge in their daily learning life, and their knowledge reserves in various aspects are relatively richer.

### 5.2 The interpretative application of machine learning model is good

In this study, we use the XGBoost model, which is a gradient boosting algorithm that randomly sets the training data in the model construction, and finally obtains the mapping relationship between the engagement variables and the output variables through error learning, and the model fits well and predicts the outcome better than the classical regression model in the case of a large number of engagement variables. Although machine learning models are considered to be black boxes to some extent, and the so-called “learning” process is unknowable, model-agnostic *a posteriori* interpretation method such as SHAP have been developed in recent years, which use comprehensible sets of rules and generate interpretable symbolic descriptions to obtaining an interpretation of the model.

In addition to global interpretation, information about the localization of the model is also of interest to this study, which is an often overlooked part of many studies using machine learning models for data analysis (Gabriel et al., 2018; Güre et al., 2020; Martínez-Abad et al., 2020). The main approach taken in this study is the analysis of SHAP values, which is an estimation method that is based on a tree model that can be based on the model to be interpreted for a specific sample or a number of samples (populations) to explore the impact of the characteristic variables at the level of the individual as well as the population, presenting the amount of the specific impact of each characteristic variable, i.e., the SHAP value. In this part of the analysis, two samples and a group with a sample size of 100 were selected to illustrate that localized explanations such as these are necessary in teaching practice, which is also in line with the need for personalized teaching in the context of modern research in educational psychology based on artificial intelligence. Teaching is carried out in the classroom as a unit, the object of the study is the whole, but the implementation of the research objectives need to be at the level of the sample or subgroups, and the characteristics or variables in different individuals, groups on the effects of the differences, a more specific understanding of the impact of these samples can better guide the direction of teaching action in order to make positive changes. At the same time, this is in line with the trend to shift research from “variable-oriented” studies of averages to “person-oriented” studies of more focused subgroups.

### 5.3 Limitation and future direction

This study has already achieved some degree of satisfactory results by applying the XGboost model, but there can still exist research space for further in-depth analysis.

On the one hand, there are limitations on data sources. This study mainly relies on the PISA2018 dataset, the choice of feature variables is limited, at the same time, the Chinese cities participating in PISA2018 are only four provinces and cities, this study is a study for the analysis of secondary data, and we are unable to make additions to the data samples from that year, which is a limitation of our article, we will also validate the results of the data on a larger scale if more samples can be collected later. In addition, the questionnaire data received varying degrees of influence from response styles and so on, and relying solely on late data cleaning is prone to cause unknowable data bias in the construction of the model. What’s more, due to the focus of the research question and time constraints, this study focuses on the independent variable which is a more representative student characteristic-reading input among the factors related to reading literacy, and in the future, we can look for the characteristics of reading literacy that affect scientific literacy the most by using more variables related to reading.

On the other hand, the algorithms used in the study are relatively homogeneous, and there are many GBRT integration algorithms, such as LightGBM, which can be further compared and investigated in order to obtain a more comprehensive grasp of the application of machine modeling to large-scale international education assessment data.

## Data availability statement

The original contributions presented in the study are included in the article/Supplementary material, further inquiries can be directed to the corresponding author.

## Author contributions

CC: Conceptualization, Data curation, Formal analysis, Methodology, Software, Validation, Visualization, Writing – original draft, Writing – review & editing. TZ: Conceptualization, Data curation, Methodology, Validation, Visualization, Writing – review & editing. TX: Conceptualization, Funding acquisition, Supervision, Validation, Visualization, Writing – review & editing.
