# Supplementary material for: The effect of reading engagement on scientific literacy – an analysis based on the XGBoost method
Source: Front Psychol. 2024 Feb 14;15:1329724. doi: 10.3389/fpsyg.2024.1329724 (PMC10899671; doi:10.3389/fpsyg.2024.1329724)
Supplement: Supplementary file 1 [file Data_Sheet_1.docx]

Supplementary Material

# Supplementary Data

The questionnaires administered to students could be find online: <https://www.oecd.org/pisa/data/2018database/>. The items analyzed in this study listed as following:

ST160: How much do you agree or disagree with these statements about reading? (Please take into account diverse kinds of reading material, such as books, magazines, newspapers, websites, blogs, emails…)

Options: Strongly disagree – Disagree – Agree – Strongly Agree

ST160Q01: I read only if I have to.

ST160Q02: Reading is one of my favourite hobbies.

ST160Q03: I like talking about books with other people.

ST160Q04: For me, reading is a waste of time.

ST160Q05: I read only to get information that I need.

ST164: Reading task: You have to understand and remember the information in a text.

How do you rate the usefulness of the following strategies for understanding and memorising the text?

Options: Not useful at all (1) – (2) – (3) – (4) – (5) – Very useful (6)

ST164Q01: I concentrate on the parts of the text that are easy to understand.

ST164Q02: I quickly read through the text twice.

ST164Q03: After reading the text, I discuss its content with other people.

ST164Q04: I underline important parts of the text.

ST164Q05: I summarise the text in my own words.

ST164Q06: I read the text aloud to another person.

ST165: Reading task: You have just read a long and rather difficult two-page text about fluctuations in the water level of a lake in Africa. You have to write a summary.

How do you rate the usefulness of the following strategies for writing a summary of this two-page text?

Options: Not useful at all (1) – (2) – (3) – (4) – (5) – Very useful (6)

ST165Q01: I write a summary. Then I check that each paragraph is covered in the summary, because the content of each paragraph should be included.

ST165Q02: I try to copy out accurately as many sentences as possible.

ST165Q03: Before writing the summary, I read the text as many times as possible.

ST165Q04: I carefully check whether the most important facts in the text are represented in the summary.

ST165Q05: I read through the text, underlining the most important sentences. Then I write them in my own words as a summary.

ST166: Reading Task: You have received a message in your inbox from a well-known mobile phone operator telling you that you are one of the winners of a smartphone. The sender asks you to click on the link to fill out a form with your data so they can send you the smartphone.

In your opinion, how appropriate are the following strategies in reaction to this email?

Options: Not appropriate at all (1) – (2) – (3) – (4) – (5) – Very appropriate (6)

ST166Q01: Answer the email and ask for more information about the smartphone

ST166Q02: Check the sender’s email address

ST166Q03: Click on the link to fill out the form as soon as possible

ST166Q04: Delete the email without clicking on the link

ST166Q05: Check the website of the mobile phone operator to see whether the smartphone offer is mentioned

ST167: How often do you read these materials because you want to? (Please take into account reading on paper and on digital devices.) (Please select one response in each row.)

ST167Q01: Magazines

ST167Q02: Comic books

ST167Q03: Fiction (novels, narratives, stories)

ST167Q04: Non-fiction books (informational, documentary)

ST167Q05: Newspapers

ST175: About how much time do you usually spend reading for enjoyment? (Please take into account diverse kinds of reading, such as books, magazines, newspapers, websites, blogs, emails…) (Please select one response.)

ST175Q01: I do not read for enjoyment

ST175Q02: 30 minutes or less a day

ST175Q03: More than 30 minutes to less than 60 minutes a day

ST175Q04: 1 to 2 hours a day

ST175Q05: More than 2 hours a day

ST176: How often are you involved in the following reading activities? (Please select one response in each row. If you don’t know what the activity is, please select “I don’t know what it is.”)

Options: I don’t know what it is – Never or almost never – Several times a month – Several times a week - Several times a day

ST176Q01: Reading emails

ST176Q02: <Chat on line> (e.g. <Whatsapp®>, <Messenger®>)

ST176Q03: Reading online news

ST176Q05: Searching information online to learn about a particular topic

ST176Q06: Taking part in online group discussions or forums

ST176Q07: Searching for practical information online (e.g. schedules, events, tips, recipes)
